# The SPICE Modeling of a Radiation Sensor Based on a MOSFET with a Dielectric HfO_2_/SiO_2_ Double-Layer

**DOI:** 10.3390/s25020546

**Published:** 2025-01-18

**Authors:** Miloš Marjanović, Stefan D. Ilić, Sandra Veljković, Nikola Mitrović, Umutcan Gurer, Ozan Yilmaz, Aysegul Kahraman, Aliekber Aktag, Huseyin Karacali, Erhan Budak, Danijel Danković, Goran Ristić, Ercan Yilmaz

**Affiliations:** 1 Department of Microelectronics, Faculty of Electronic Engineering, University of Niš, 18000 Niš, Serbia; sandra.veljkovic@elfak.ni.ac.rs (S.V.); nikola.i.mitrovic@elfak.ni.ac.rs (N.M.); danijel.dankovic@elfak.ni.ac.rs (D.D.); goran.ristic@elfak.ni.ac.rs (G.R.); 2 Center of Microelectronic Technologies, Institute of Chemistry, Technology and Metallurgy, University of Belgrade, 11000 Belgrade, Serbia; stefan.ilic@nanosys.ihtm.bg.ac.rs; 3 Faculty of Arts and Sciences, Bolu Abant Izzet Baysal University, 14280 Bolu, Turkey; gurerumutcan@gmail.com (U.G.); ozan84yilmaz@gmail.com (O.Y.); aktag_a@ibu.edu.tr (A.A.); karacali_h@ibu.edu.tr (H.K.); erhan@ibu.edu.tr (E.B.); prof.dr.ercan.yilmaz@gmail.com (E.Y.); 4 Department of Physics, Faculty of Arts and Sciences, Bursa Uludag University, 16059 Bursa, Turkey; aysegulk@uludag.edu.tr

**Keywords:** SPICE model, RADFET, high-k materials, radiation sensor, electrical simulation

## Abstract

We report on a procedure for extracting the SPICE model parameters of a RADFET sensor with a dielectric HfO_2_/SiO_2_ double-layer. RADFETs, traditionally fabricated as PMOS transistors with SiO_2_, are enhanced by incorporating high-k dielectric materials such as HfO_2_ to reduce oxide thickness in modern radiation sensors. The fabrication steps of the sensor are outlined, and model parameters, including the threshold voltage and transconductance, are extracted based on experimental data. Experimental setups for measuring electrical characteristics and irradiation are described, and a method for determining model parameters dependent on the accumulated dose is provided. A SPICE model card is proposed, including parameters for two dielectric thicknesses: (30/10) nm and (40/5) nm. The sensitivities of the sensors are 1.685 mV/Gy and 0.78 mV/Gy, respectively. The model is calibrated for doses up to 20 Gy, and good agreement between experimental and simulation results validates the proposed model.

## 1. Introduction

MOSFET (Metal–Oxide–Semiconductor Field-Effect Transistor) radiation sensors, also known as RADFET (Radiation-Sensitive FET), are semiconductor-based sensors employed for the detection and measurement of ionizing radiation [1,2,3]. When exposed to radiation, additional electron–hole pairs in the oxide are generated. The initial recombination of some electrons and holes occurs. Unrecombined electrons leave the oxide, resulting in the formation of a fixed positive charge [4,5,6]. The positive charge is also trapped at the Si/SiO_2_ interface. The fixed charge affects the change in the threshold voltage of the transistor and transconductance, i.e., the carrier mobility and drain leakage current. These parameter changes are proportional to the total accumulated dose of ionizing radiation [7,8].

RADFETs can be used as passive (without bias during irradiation) or active (with bias during irradiation) integrated dosimeters [9,10]. The reading of these sensors is possible instantly, without data loss. When reading RADFETs, the pins are typically configured such that the transistor operates in the saturation region, and the gate and drain are shorted, while the source is connected to the ground. Reading the sensor means measuring the transfer characteristic of the RADFET in the saturation region by varying the voltage between the gate and source V_GS_ and reading the drain current I_D_, or, vice versa, by driving a current I_D_ and measuring V_GS_. Through the further processing of the transfer characteristic of the RADFET, it is possible to indirectly determine the total accumulated dose.

The advantages of using RADFETs are their small chip area, their very low or zero power consumption, the technology being suitable for connection to microcontroller circuits, and the sensors being sensitive to various forms of ionizing radiation [11,12], such as electrons, X-rays, and protons. Research has shown that the polarization of the transistor during irradiation affects the sensitivity, with unbiased transistors demonstrating higher sensitivity than those that are biased during irradiation [13,14,15]. The thickness of the dielectric layer in traditional RADFETs with the SiO_2_ dielectric affects the sensitivity of the sensor, as a thicker oxide layer results in more significant changes to the threshold voltage with an increasing radiation dose [16,17].

The challenge in the application of RADFETs is the fading effect, which refers to the gradual loss of information regarding the accumulated dose over time. Studies have shown that unbiased RADFETs have a less pronounced fading effect compared to biased transistors [18]. It was shown that the average fading for unbiased RADFET samples with a 400 nm thick SiO_2_ is 4% after 5 days and 5% after one month, which are lower rates compared to biased samples, where the average fading is 13.5% after 5 days and 20% after 21 days. Bhat et al. [19] reported that the annealing or long-term fading of 400 nm RADFETs based on SiO_2_ is less than 1% at 100 °C over a period of 1000 h. Although RADFET parameters are temperature-dependent, this issue can be mitigated by operating the device at the Zero-Temperature Coefficient (ZTC) point during the readout process [18,20]. RADFETs are widely used for both low-dose and high-dose detection in various applications, including personal dosimetry, medical diagnostics, radiotherapy, physical laboratories, and space research [21,22,23,24,25,26,27,28,29,30]. The main disadvantage in the application of RADFETs is their inability to be re-used, as well as poor sensitivity for extremely low doses. A group of authors from Tyndall, formerly the NMRC (Cork, Ireland), has advanced the capabilities of RADFETs for dosimetry in diverse applications while addressing key limitations. Kelleher et al. explored the re-use of PMOS dosimeters through annealing but identified reduced sensitivity during subsequent irradiations due to neutral electron traps, which create a “memory effect” [31]. Later, a stacked RADFET configuration was introduced to enhance sensitivity for low-dose applications, but challenges such as increased output voltage, potentially exceeding the breakdown limits of individual devices, and amplified drift and fading effects were noted [32,33]. Further work by Kelleher et al. examined on-chip annealing techniques using poly-resistor heaters, demonstrating promising results for in situ recovery and extended use in space-constrained environments [34].

High-k materials have emerged as an alternative in modern MOS technologies due to the increasing demand for an equivalent oxide thickness (EOT) of less than 1 nm due to device scaling. However, the interface between high-k materials and silicon contains many structural defects caused by lattice mismatch, leading to increased interface traps. A drawback of these materials is the need for a SiO_2_ buffer layer between the high-k material and the silicon substrate, which increases the EOT and eliminates the chance of achieving an EOT well below 1 nm [35]. In a few papers [36,37], the authors demonstrated that high-k materials are utilized in the latest FinFET and nanowire FET technologies, offering advantages such as lower leakage currents and improved thermal stability compared to traditional SiO_2_. Additionally, high-k materials are attractive in sensor technologies. A comprehensive review conducted by our team [38] provides an overview of high-k materials used in sensor fabrication, such as RADFETs, oxide thicknesses, dose values in experiments, and the corresponding sensitivities of sensors made from these materials. Notably, the highest sensitivity of 107 mV/Gy was achieved using Er_2_O_3_ with a thickness of 254 nm [9], while a slightly lower sensitivity of approximately 28 mV/Gy was obtained with Yb_2_O_3_, which had a thickness of 114 nm [39]. In another study [40], HfO_2_ with a thickness of 180 nm was employed as a high-k material, resulting in a sensitivity of less than 2 mV/Gy. The fading effect is also present in high-k RADFETs. Kaya [41] demonstrated that RADFETs with Er_2_O_3_ without bias during irradiation exhibit fading of up to 20% after 14 days. The higher percentage of fading observed in RADFETs with high-k dielectrics is attributed to charge trapping both in the oxide and at the Si–dielectric interface, resulting in higher recombination. Additionally, high-k dielectrics have a greater number of shallow traps compared to SiO_2_ [41], which further contributes to this effect.

Computer-aided design (CAD) models are essential in the development and implementation of electronic devices, facilitating all stages from design to fabrication and the simulation of device behavior within a complex system. Analytical models help in developing CAD models. For example, Andreev et al. [42] propose a model that enables the quantitative analysis of charge effects in radiation MOS sensors under the concurrent influence of ionizing radiation and the high-field tunnel injection of electrons. Technology computer-aided design (TCAD) models are widely used to simulate and optimize device geometry before fabrication [43,44]. On the other hand, SPICE (Simulation Program with Integrated Circuit Emphasis) models are employed to simulate the effects of ionizing radiation on components and electronic systems. For example, Pesic-Brdjanin [45] introduces a method for incorporating ionizing radiation effects into SPICE models of MOS transistors and FinFETs. This method utilizes an auxiliary diode circuit to derive values for surface potential and calculates the time-dependent voltage correction due to trapped charge concentration. In addition, Mebrahtu et al. [46] developed behavioral SPICE models to assess the contribution of photodiodes and static small-signal NMOS transistors to the increase in the dark current of CMOS active-pixel sensors during irradiation. Kerdpradist et al. [47] investigate the threshold voltages and drain currents of large and short n-channel MOSFETs during irradiation using a SPICE Level 3 model. Furthermore, Zebrev et al. [48] propose an extraction procedure for the SPICE parameters of the equivalent parasitic transistor, which facilitates the transfer of parameters from physical models to circuit-level simulations using CAD tools. Our research group previously published a SPICE model for RADFETs using SiO_2_ as the dielectric [49]. This model was effectively employed to simulate RADFETs with oxide thicknesses ranging from 40 nm to 300 nm.

This paper presents a procedure for extracting SPICE model parameters for a radiation sensor based on a MOSFET with a double-layer dielectric comprising HfO_2_ and SiO_2_. The paper is structured as follows: Section 2 outlines the sensor fabrication process, the experimental setup for measuring its electrical characteristics, and the irradiation setup. Section 3 describes the methods for extracting SPICE model parameters that depend on the radiation dose, including the extraction of threshold voltage, transconductance parameters, and the transistor channel length modulation factor. Section 4 presents the results, defines calibration functions for the extracted parameters across all tested samples, establishes the SPICE model card, and validates the proposed model. Finally, Section 5 provides a summary of the conclusions drawn from this research.

## 2. Materials and Methods

**Figure 1:**
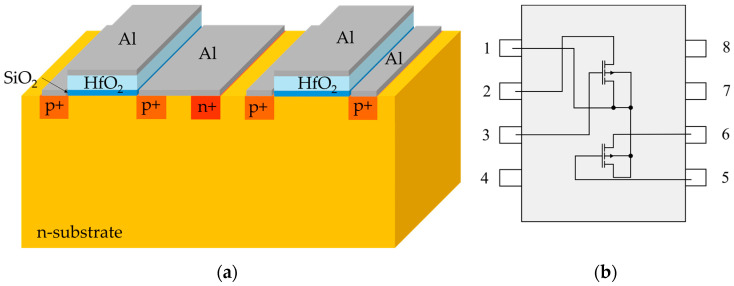
(**a**) Cross-sectional view of the MOSFET structure with the high-k dielectric; (**b**) bonding diagram of the radiation sensor.

The fabrication process of MOSFET radiation sensors begins with using 6-inch n-type Si (100) wafers (2–4 Ωcm, one side polished, 500 µm thick), which undergo an RCA cleaning procedure to eliminate any potential contaminants. To grow the required field oxide layer (1.5 μm) for the subsequent lithography process, both wet and dry thermal oxidation methods are applied. Following the first lithography step, etching and doping are performed to define the source/drain regions. The MOSFETs are designed with a channel width-to-length ratio of 600/50. In the second lithography step, after etching, a gate oxide layer is deposited. The gate oxide comprises two layers: SiO_2_ and a high-k material, HfO_2_. The purity of Hf powder is 99.999%. The thicknesses of the HfO_2_/SiO_2_ layers are set at (30/10) nm and (40/5) nm, respectively, and are measured using ellipsometry. It should be noted that these oxide thicknesses were obtained by analyzing the results of TCAD simulations prior to fabrication [43]. In the third lithography step, etching is again performed, followed by the deposition of aluminum metal contacts using a DC magnetron sputtering system. Each chip contains two distinct MOSFET structures. In one MOSFET, the source and bulk terminals are internally connected, while in the other, the source and bulk terminals are separated. The cross-sectional view of the MOSFET structure with the high-k dielectric is shown in Figure 1a, while the bonding diagram of the radiation sensor is presented in Figure 1b.

**Figure 2:**
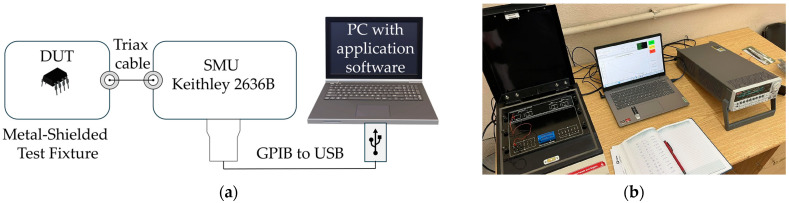
(**a**) Block diagram of the measurement setup; (**b**) measurement setup in the TENMAK lab.

The current–voltage (transfer and output) characteristics of the device under test (DUT), which is the radiation sensor, were measured using the setup shown in the block diagram in Figure 2a. The DUT was placed inside a metal-shielded test fixture to minimize external interference and was connected to a Keithley 2636B (Santa Rosa, CA, USA) Source-Measure Unit (SMU) via a triaxial cable. The SMU was responsible for sourcing the current (in a range from 1 µA to 60 µA) and measuring the voltage across the DUT. During the measurements, the drain and gate terminals of the MOSFETs were shorted, so the operation of the transistor in the saturation region was ensured. The SMU was connected to a computer through a GPIB to USB interface, allowing seamless communication for control and data acquisition. To automate the measurement process, a custom software application was developed using C#, was developed using Visual Studio 2019 (Community edition), which facilitated SMU control and real-time data collection. This software enhanced the efficiency of the measurements by enabling precise automation and minimizing human error during testing. The limitation of this measurement setup is the inability to record the subthreshold current–voltage characteristics of the transistor, which is not crucial for creating an accurate SPICE model and therefore is not addressed in this paper. A photograph of the measurement setup is shown in Figure 2b.

**Figure 3:**
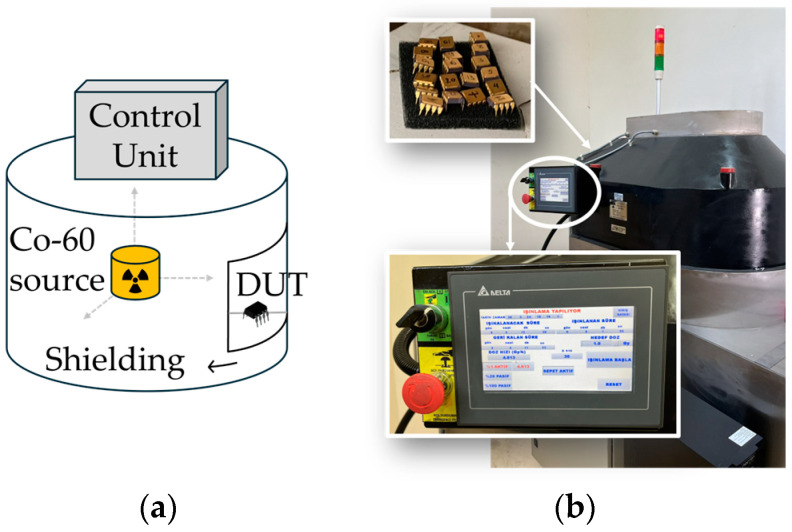
(**a**) Block diagram of the radiation source setup; (**b**) radiation setup in the TENMAK lab.

The sensors were irradiated at the TENMAK (Turkish Energy, Nuclear, and Mineral Research Agency) [50] facility using a ^60^Co radiation source, with doses ranging from 1 to 20 Gy and a dose rate of 4.813 Gy/h in an air environment. During irradiation, the source was kept stable, and to ensure homogeneous dose distribution, the sample basket was rotated. The limitation of this setup is the inability to bias samples during irradiation due to the design of the radiation source itself. The radiation source setup is shown in the block diagram in Figure 3a, while a photograph of the laboratory is presented in Figure 3b. The DUTs were measured both before and during irradiation, at accumulated doses of 1, 2.5, 5, 7.5, 10, 15, and 20 Gy. During irradiation, the sensors were not biased. These measurements were crucial for extracting the parameters of the SPICE model used for radiation sensor simulation, providing insights into the sensor’s behavior under varying radiation doses.

## 3. SPICE Model Parameter Extraction

SPICE models provide a comprehensive framework for simulating the electrical behavior of MOSFETs under various operating conditions. The extraction of key parameters, such as threshold voltage and transconductance, involves analyzing the device’s current–voltage characteristics and fitting experimental data to theoretical models. Accurate parameter extraction is essential for predicting device performance, ensuring that simulations align with real-world measurements. This process typically includes measurements of the device’s transfer and output characteristics, conducted using specialized techniques, such as linear extrapolation, and optimization algorithms for parameter extraction [51,52,53,54].

SPICE-like simulators calculate the drain current *I_D_* of the MOSFET transistor according to the formula [55]

(1) $$I_{D}=\left\{\begin{array}{c} 0,V_{GS}<V_{T}\\ \frac{KP}{2}\frac{W}{L}\left(1+LAMBDA\cdot V_{DS}\right)\left(2\left(V_{GS}-V_{T}\right)-V_{DS}\right)V_{DS},\;V_{DS}<V_{GS}-V_{T}\\ \frac{KP}{2}\frac{W}{L}\left(1+LAMBDA\cdot V_{DS}\right){\left(V_{GS}-V_{T}\right)}^{2},V_{DS}>V_{GS}-V_{T} \end{array}\right.$$

where *V_GS_* is the gate–source voltage, *V_T_* is the threshold voltage, *V_DS_* is the drain–source voltage, *L* is the channel length, *W* is the channel width, *KP* is the SPICE transconductance coefficient, and *LAMBDA* is the channel length modulation factor. It should be noted that the threshold voltage *V_T_* in SPICE is implemented as a complex function of the following parameters—VTO—zero-bias threshold voltage; GAMMA—bulk threshold parameter; and PHI—surface potential—where the dominant value is VTO, which is extracted from the experimental data, and other parameters take default values [56].

### 3.1. Zero-Bias Threshold Voltage

The threshold voltage *V_T_* of the MOSFET, operating in the saturation region (*V_DS_* > *V_GS_* − *V_T_*), is extracted from the transfer current–voltage characteristics by utilizing the dependence:

(2) $$I_{D}=k{\left(V_{GS}-V_{T}\right)}^{2},$$

where *k* is a constant that depends on the device geometry and process parameters (*µ*—carrier’s mobility; *ε*_0_—dielectric permittivity of the vacuum; *k_ox_*—permittivity of the gate oxide material; *t_ox_*—oxide thickness):

(3) $$k=\frac{\mu \cdot \varepsilon_{0}\cdot k_{ox}\cdot W}{2\cdot t_{ox}\cdot L}.$$

By plotting √*I_D_* versus *V_GS_*, the threshold voltage *V_T_* can be determined as the *V_GS_* value where the extrapolated linear portion of the curve intersects the x-axis. The obtained value is taken for the SPICE parameter VTO—zero-bias threshold voltage—and it is dose-dependent.

### 3.2. Transconductance Coefficient

The transconductance *g_m_* of a MOS transistor is defined as the variation in the drain current with respect to a change in the gate voltage, while keeping the drain voltage constant:

(4) $$g_{m}=\frac{dI_{D}}{dV_{GS}},\;V_{DS}=const.$$

By differentiating Equation (2), the transconductance value in the saturation region is obtained:

(5) $$g_{m}=2k\left(V_{GS}-V_{T}\right).$$

The value of the SPICE parameter *KP*, the transconductance coefficient, is obtained by determining the slope coefficient *k* defined by Equation (5):

(6) $$KP=\frac{k\cdot L}{W}.$$

The *KP* parameter includes the carrier mobility in the transistor channel and gate oxide parameters, and this parameter depends on the total accumulated dose.

### 3.3. Channel Length Modulation Factor

The channel length modulation factor—*LAMBDA*—refers to an increase in the depletion layer between the drain and source as the drain voltage is increased. The parameter *LAMBDA* is calculated by selecting two points on the drain current axis and their corresponding *V_DS_* voltage values from the output characteristics curve, keeping the gate voltage *V_GS_* constant [57]:

(7) $$LAMBDA=\frac{I_{D2}-I_{D1}}{I_{D1}V_{DS2}-I_{D2}V_{DS1}}.$$

The *LAMBDA* parameter depends on the radiation dose.

## 4. Results and Discussion

**Figure 4:**
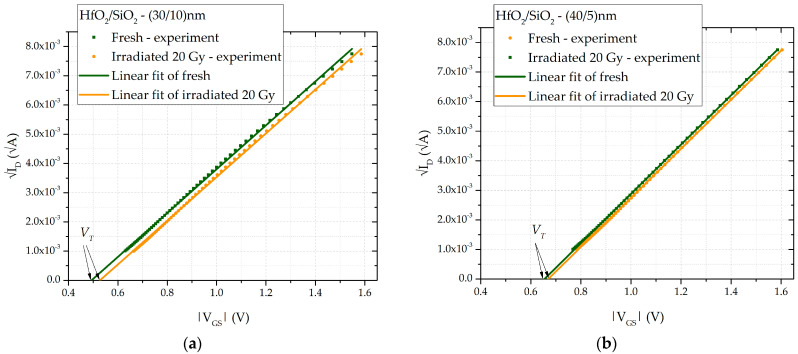
Transfer characteristic showing the square root of the drain current as a function of voltage, |V_GS_|, for double-layer dielectric HfO_2_/SiO_2_ RADFETs of (**a**) (30/10) nm and (**b**) (40/5) nm.

**Figure 5:**
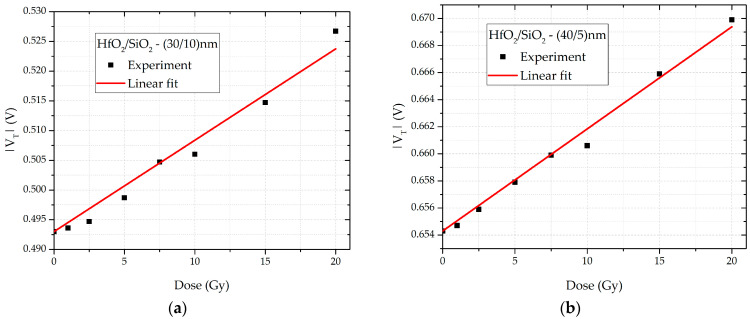
Dependence of the absolute value of the threshold voltage on the accumulated dose for double-layer dielectric HfO_2_/SiO_2_ RADFETs of (**a**) (30/10) nm and (**b**) (40/5) nm.

The experimentally measured transfer characteristic is presented as the square root of the drain current (√*I_D_*) plotted as a function of the absolute value of the gate–source voltage (|*V_GS_*|), as this relationship is linear. The threshold voltage (*V_T_*) was determined using the linear extrapolation method, which involves identifying the voltage value *V_GS_* where the fitted straight line intersects the *x*-axis. Figure 4a illustrates this method for samples with a dielectric layer thickness of (HfO_2_/SiO_2_) = (30/10) nm, while Figure 4b shows the same method for samples with (HfO_2_/SiO_2_) = (40/5) nm. To enhance clarity, two sets of data are presented: one for unirradiated (fresh) samples and the other for irradiated samples, which have accumulated a total dose of 20 Gy. Through the use of this method, the threshold voltage was determined for all samples and doses considered. The dependence of the absolute value of the threshold voltage on the accumulated dose is shown in Figure 5. For small doses, this dependence follows a linear relationship:

(8) $${|V}_{T}|=a_{1}+b_{1}\cdot DOSE,$$

where *a*_1_ and *a*_2_ are the fitting constants, and *DOSE* represents the accumulated dose. The obtained values of the fitting constants for all samples are summarized in Table 1. It is important to note that studies have shown this dependence to be linear for small doses up to 100 Gy [10,13]. Radiation creates electron–hole pairs, which undergo recombination. Unrecombined electrons leave the dielectric, resulting in a positive fixed charge both in the dielectric and at the silicon interface. The increased concentration of hole traps leads to a shift in the threshold voltage. In PMOS transistors, as in this study, the threshold voltage increases in absolute value with the dose.

**Figure 6:**
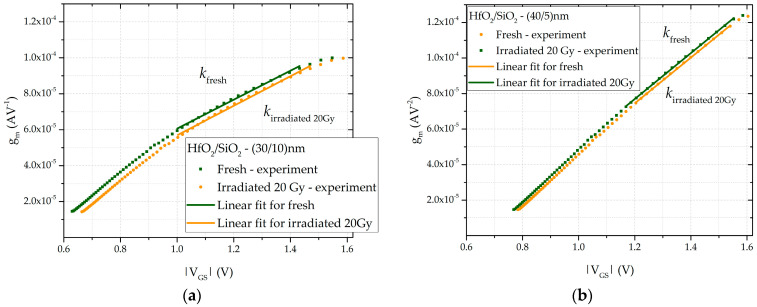
Transconductance as a function of voltage, |*V_GS_*|, for double-layer dielectric HfO_2_/SiO_2_ RADFETs of (**a**) (30/10) nm and (**b**) (40/5) nm.

**Figure 7:**
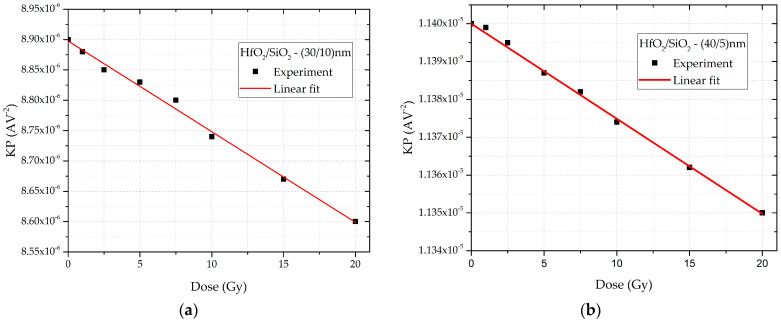
Dependence of the SPICE model parameter KP on the accumulated dose for double-layer dielectric HfO_2_/SiO_2_ RADFETs of (**a**) (30/10) nm and (**b**) (40/5) nm.

Based on the experimental data, the transistor transconductance was calculated. The dependence of the transconductance on the absolute value of the gate–source voltage for both types of samples is shown in Figure 6. Curves for unirradiated and irradiated samples, with a total accumulated dose of 20 Gy, are presented to enhance clarity. Through the determination of the slope coefficient of the linear region, and the use of the known dimensions of the transistor, the SPICE model parameter can be derived from Equation (6). The coefficient is obtained by selecting the portion of the transfer characteristic above the “knee” of the curve. The dependence of the parameter on the accumulated dose is shown in Figure 7. This relationship is described by a linear function:

(9) $$KP=a_{2}-b_{2}\cdot DOSE,$$

where *a_2_* and *b_2_* are the fitting constants. The values of these constants for all transistors are provided in Table 1. Since this parameter is directly proportional to carrier mobility and inversely proportional to the effective gate oxide thickness, its value decreases with increasing dose. Radiation-induced traps at the oxide interface contribute to the reduction in carrier mobility in the transistor channel.

**Figure 8:**
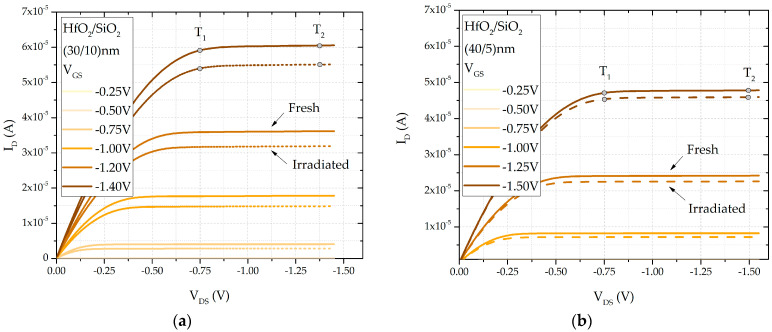
*I_D_-V_DS_* characteristic of double-layer dielectric HfO_2_/SiO_2_ RADFETs of (**a**) (30/10) nm and (**b**) (40/5) nm.

The SPICE model parameter LAMBDA can be determined from the slope of the output I_D_-V_DS_ characteristic of the transistor at a constant gate–source voltage, as described previously. The measured output characteristic of the transistors, with an oxide double-layer (HfO_2_/SiO_2_) of (30/10) nm and (40/5) nm, both before and after irradiation, with marked points T1 and T2 for determining the parameter *LAMBDA*, is shown in Figure 8. The dependence of the parameter LAMBDA on the accumulated dose is given by

(10) $$LAMBDA=a_{3}-b_{3}\cdot DOSE,$$

where *a_3_* and *b_3_* are the fitting constants, the values of which are provided in Table 1. It can be concluded that while changes in this parameter, *LAMBDA*, due to irradiation exist, they are not highly significant. It should be noted that the setup for measuring these characteristics differs from previous measurements; an SMU is used, but the gate and drain of the transistor are not short-circuited. Instead, a fixed voltage is applied to the gate, a constant current ranging from 1 µA to 60 µA is injected into the transistor, and the voltage *V_DS_* is measured.

Based on the extracted dependencies of various SPICE parameters of the MOS transistor as a function of the accumulated dose, a SPICE model card was created. In addition to the dose-dependent parameters, geometric parameters, such as channel length and width, are also defined. The parameter *TPG* = 0 indicates that the transistor uses an aluminum gate. Parameters not explicitly defined in the model card, but when used in the calculations, are assigned default values. The user defines the dose value by specifying the DOSE parameter using the SPICE command PARAM. Furthermore, the user can select whether to run the simulation with a transistor having a double-layer gate dimension of (30/10) nm or (40/5) nm by setting the TYPE parameter to 1 or 2, respectively. The corresponding model card is presented below:

.MODEL RADFET PMOS VTO={if(TYPE==1,-0.493-(1.54e-3*DOSE),-0.65433-(7.54E-4*DOSE))}
+KP={if(TYPE==1,8.897e-6-(1.493e-8*DOSE),1.14E-5-(2.511E-9*DOSE))} L=50e-6 W=600e-6
+TPG=0 LAMBDA={if(TYPE==1,3.901E-2-(2.165E-4*DOSE),2.0115E-2-(1.8575E-4*DOSE))}

**Figure 9:**
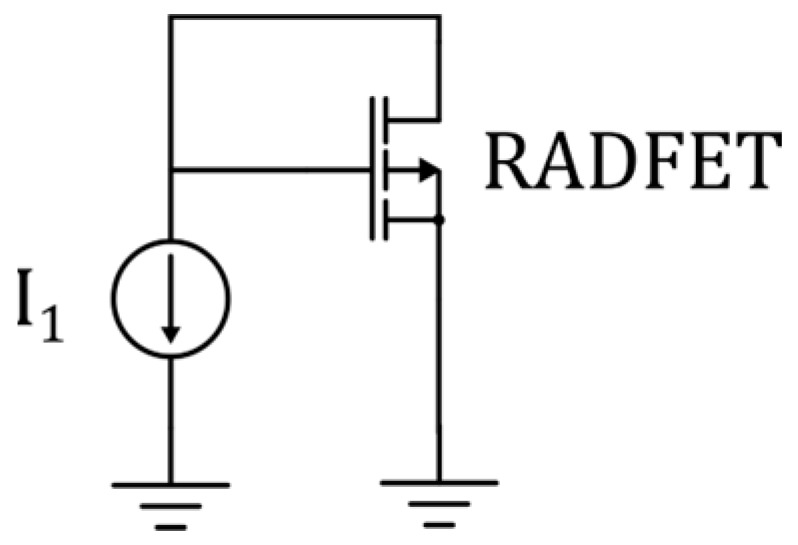
Simulation circuit.

The simulations were carried out using the open-source tool LTspice [58]. An electrical schematic was created to replicate the experimental setup used for measuring the transistor characteristics, as shown in Figure 9. In this setup, the gate and drain of the transistor are short-circuited to ensure that the transistor operates in the saturation region, with the source grounded. A current *I*_1_ = *I_D_* in the range of 1 µA to 60 µA is passed through the transistor, and the voltage *V_GS_* = *V_DS_* is measured.

**Figure 10:**
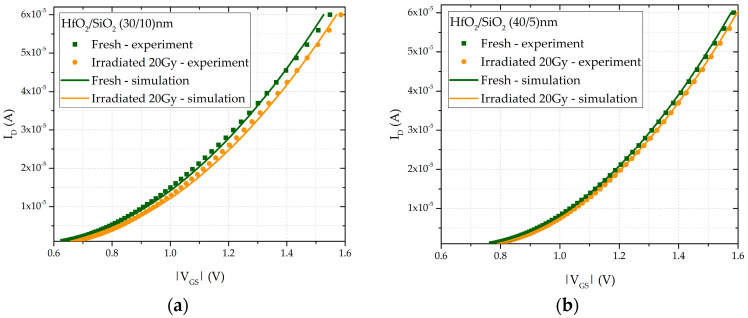
Experimental and simulated transfer characteristics for double-layer dielectric HfO_2_/SiO_2_ RADFETs of (**a**) (30/10) nm and (**b**) (40/5) nm.

The comparison of experimental and simulated transfer characteristics for both types of transistors is shown in Figure 10. A good agreement between the experimental data and the simulation results can be observed, validating the accuracy of the proposed model. For the PMOS transistors considered as radiation sensors, the sensitivity was determined to be 1.685 mV/Gy for the transistor with an oxide thickness of (30/10) nm, and 0.78 mV/Gy for the transistor with an oxide thickness of (40/5) nm. As the thickness of the HfO_2_ layer increases, the sensor sensitivity decreases. This decrease in sensitivity may be attributed to the negative charge trap centers becoming comparable in number to the positive charge trap centers. When compared to similar sensors reported in the literature [9,39,40], the observed trends are consistent. The primary advantage of the multi-layer RADFET design with high-k dielectrics over the conventional SiO_2_-based design lies in its sufficiently high sensitivity, which is achieved despite the much thinner oxide layers. Commercial RADFETs [59] with a 100 nm thick SiO_2_ dielectric exhibit a sensitivity of 1.5 mV/Gy, whereas those with a 400 nm thick SiO_2_ dielectric show a sensitivity ranging from 55 to 65 mV/Gy, and those with a 1 µm thick SiO_2_ dielectric have sensitivities between 135 and 324 mV/Gy. Through a comparison of the sensitivities of commercial RADFETs with SiO_2_ dielectrics to those of RADFETs with HfO_2_/SiO_2_ dielectrics, the advantage of using high-k dielectrics becomes evident. A reduction in the SiO_2_ dielectric thickness leads to a decrease in RADFET sensitivity, which in turn results in higher leakage currents and increased power dissipation [60]. This effect contrasts with the behavior of high-k dielectrics, which maintain sufficiently high sensitivity while minimizing leakage currents and power loss.

**Figure 11:**
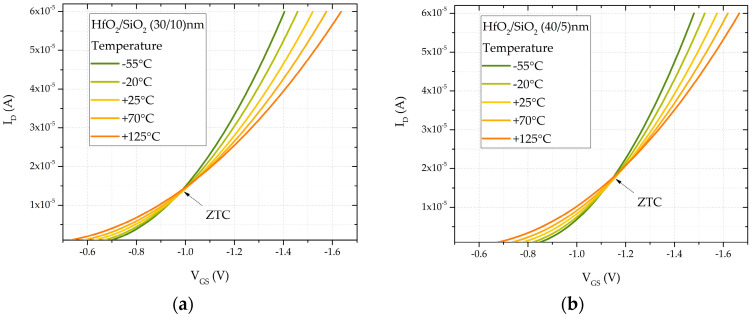
Simulated transfer characteristics at different temperatures for double-layer dielectric HfO_2_/SiO_2_ RADFETs of (**a**) (30/10) nm and (**b**) (40/5) nm.

This SPICE sensor model can be used in the design of dosimeter electronic circuits, which, in addition to the sensor, typically include data processing circuits, often based on a microcontroller. In dosimeter circuit design, a controlled current is forced through the transistor, and the resulting voltage is measured, representing the threshold voltage for further data processing. The simulated current–voltage characteristics at different temperatures for non-irradiated (fresh) sensors, based on this configuration, are presented in Figure 11. The .TEMP SPICE command is used for temperature simulation. To optimize temperature compensation, the recommended current value is determined by the Zero-Temperature Coefficient (ZTC) point, where the temperature effect is minimized. Based on the simulation results, for samples with dielectric thicknesses (HfO_2_/SiO_2_) = (30/10) nm, the optimal current for sensor operation is 14 µA, while for samples with (HfO_2_/SiO_2_) = (40/5) nm, the optimal value is 18 µA, minimizing temperature sensitivity.

**Figure 12:**
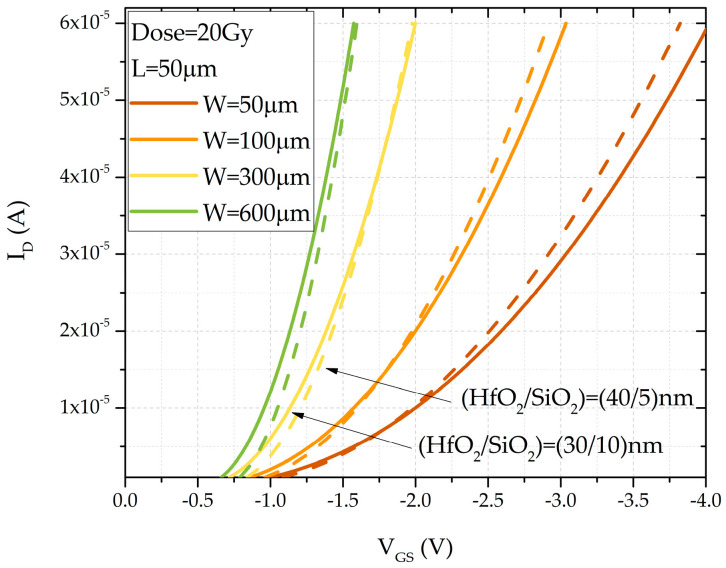
Simulated transfer characteristics for double-layer dielectric HfO_2_/SiO_2_ RADFETs with different channel widths.

Through the use of the model, the sensor characteristics can be estimated prior to fabrication, allowing for the optimization of the transistor’s geometric parameters. The model has been calibrated to the experimental values of fabricated sensors with the following channel dimensions: (W/L) = (600/50) µm. Given the ongoing trend in microelectronics toward reducing transistor dimensions, the model has been applied to evaluate sensor characteristics with different channel widths, while keeping the channel length fixed at L = 50 µm for both considered dielectric thicknesses. Figure 12 shows the simulation results for four channel widths—50 µm, 100 µm, 300 µm, and 600 µm—with a dose value of 20 Gy. From these results, it can be concluded that as the channel width decreases, the threshold voltage increases and the slope of the current–voltage curves changes, indicating a shift in the transconductance as the channel width varies.

However, the limitation of this model is that it is currently only applicable to the specific oxide thicknesses presented here. If the oxide thickness is altered, additional calibration of the model parameters would be necessary. The model can also be applied when using other high-k materials, but the extraction of the model parameter values should follow the procedure described in this paper. In our future research, we plan to investigate the fading effect in RADFETs based on HfO_2_/SiO_2_ dielectrics. Based on these experimental results, we will refine the proposed SPICE model by introducing an additional parameter to represent the recovery time, which will allow us to calculate the new threshold voltage value.

## 5. Conclusions

In this paper, a SPICE model for a radiation sensor based on a PMOS transistor with a double-layer dielectric of HfO_2_/SiO_2_ has been presented. The sensor samples were fabricated following the described procedure, and model parameters were extracted from detailed experimental measurements. The model incorporates parameters for two different dielectric thicknesses: (30/10) nm and (40/5) nm. In addition to the geometric parameters determined by the fabrication process, the model includes parameters dependent on the accumulated radiation dose, such as the threshold voltage (VTO), transconductance parameter (KP), and channel length modulation factor (LAMBDA). All these parameters show a linear dependence on the dose, up to 20 Gy. The fitting coefficients for these dependencies were determined and presented. A SPICE model card for the RADFET sensor, incorporating both oxide layer thicknesses, was developed and tested in LTspice, demonstrating good agreement with experimental results, thus confirming the model’s validity. This model can be utilized in the design of similar radiation sensors, for the optimization of RADFET geometry, and in the design and simulation of complex electronic circuits incorporating RADFETs. While changes in oxide layer thicknesses may require additional calibration, the fundamental principles of the model remain applicable.

## Figures and Tables

**Table 1:** Fitting constants for dose-dependent SPICE model parameters VTO, KP, and LAMBDA.

Parameter		HfO_2_/SiO_2_ (30/10) nm	HfO_2_/SiO_2_ (40/5) nm
*VTO*	*a*_1_	0.493	0.6543
	*b*_1_	1.54 × 10^−3^	7.54 × 10^−4^
*KP*	*a*_2_	8.897 × 10^−6^	1.14 × 10^−5^
	*b*_2_	1.493 × 10^−8^	2.511 × 10^−9^
*LAMBDA*	*a*_3_	3.901 × 10^−2^	2.0115 × 10^−2^
	*b*_3_	2.165 × 10^−4^	1.8575 × 10^−4^

## Data Availability

The data presented in this study are available on request from the corresponding author.
